# Supplementary material for: Significance of Identifying Key Genes Involved in HBV-Related Hepatocellular Carcinoma for Primary Care Surveillance of Patients with Cirrhosis
Source: Genes (Basel). 2022 Dec 10;13(12):2331. doi: 10.3390/genes13122331 (PMC9778294; doi:10.3390/genes13122331)
Supplement: Supplementary file 1 [file genes-13-02331-s001.zip › Table S1. Exploration of 120 potential DEGs in the GSE121248 dataset.pdf]

Table S1. Exploration of 120 potential DEGs in the GSE121248 dataset.

Gene symbol	Gene description	logFC	P value	Up/down
LINC01419	Long Intergenic Non−Protein Coding RNA 1419	2.084786602	1.94E−06	Up
MAGEA6	MAGE Family Member A6	2.171022861	1.13E−05	Up
PEG10	Paternally Expressed 10	2.042842120	4.82E−05	Up
ACSL4	Acyl−CoA Synthetase Long Chain Family Member 4	2.813819514	2.85E−13	Up
GPC3	Glypican 3	3.847841892	1.89E−15	Up
CTHRC1	Collagen Triple Helix Repeat Containing 1	2.337200459	2.39E−09	Up
COL15A1	Collagen Type XV Alpha 1 Chain	2.968063583	2.40E−21	Up
SPP1	Secreted Phosphoprotein 1	2.101476266	5.48E−07	Up
SULT1C2	Sulfotransferase Family 1C Member 2	2.107128429	7.96E−08	Up
AKR1B10	Aldo−Keto Reductase Family 1 Member B10	3.068006046	5.05E−09	Up
RBM24	RNA Binding Motif Protein 24	2.111902112	5.85E−12	Up
CAP2	Regulatory Protein 2	2.470035042	9.68E−26	Up
HMMR	Hyaluronan Mediated Motility Receptor	2.220242695	1.27E−20	Up
PBK	PDZ Binding Kinase	2.217813595	5.08E−16	Up
RRM2	Ribonucleotide Reductase Regulatory Subunit M2	2.248686305	4.98E−18	Up
ECT2	Epithelial Cell Transforming 2	2.038996714	4.09E−19	Up
BUB1B	BUB1 Mitotic Checkpoint Serine/Threonine Kinase B	2.049502320	2.77E−17	Up
PRC1	Protein Regulator Of Cytokinesis 1	2.085868676	4.28E−19	Up
CCNB1	Cyclin B1	2.053859931	7.24E−17	Up
TOP2A	DNA Topoisomerase II Alpha	3.261963386	8.89E−24	Up
NEK2	NIMA Related Kinase 2	2.314657830	7.55E−18	Up
CDK1	Cyclin Dependent Kinase 1	2.029493985	2.54E−16	Up
ANLN	Anillin, Actin Binding Protein	2.499189772	6.93E−19	Up
DTL	Denticleless E3 Ubiquitin Protein Ligase Homolog	2.197331263	1.05E−17	Up
RACGAP1	Rac GTPase Activating Protein 1	2.002401328	2.12E−22	Up
ASPM	Assembly Factor For Spindle Microtubules	2.859644768	4.05E−22	Up
ZIC2	Zic Family Member 2	2.045716266	1.11E−10	Up
IGF2BP3	Insulin Like Growth Factor 2 MRNA Binding Protein 3	2.238629923	3.59E−09	Up
CRNDE	Colorectal Neoplasia	2.652245521	8.79E−13	Up

	Differentially Expressed			
ROBO1	Roundabout Guidance Receptor 1	2.024942977	5.94E−15	Up
SPINK1	Serine Peptidase Inhibitor Kazal Type 1	3.656277417	6.58E−10	Up
SLCO1B3	Solute Carrier Organic Anion Transporter Family Member 1B3	−2.927220730	6.87E−10	Down
ADH4	Alcohol Dehydrogenase 4 (Class II), Pi Polypeptide	−2.121819958	2.47E−06	Down
SLC22A1	Solute Carrier Family 22 Member 1	−2.529147583	2.49E−09	Down
ESR1	Estrogen Receptor 1	−2.275245143	1.02E−14	Down
FLJ22763	Chromosome 3 Open Reading Frame 85	−2.715307483	7.61E−15	Down
LINC00844	Long Intergenic Non−Protein Coding RNA 844	−2.349710946	8.90E−07	Down
C9	Complement C9	−2.786415328	1.46E−07	Down
FAM134B	Reticulophagy Regulator 1	−2.339624903	1.85E−11	Down
THRSP	Thyroid Hormone Responsive	−2.993124985	2.11E−09	Down
TENM1	Teneurin Transmembrane Protein 1	−2.375247127	2.96E−10	Down
FOLH1B	Folate Hydrolase 1B (Pseudogene)	−2.113008154	3.54E−14	Down
BBOX1	Gamma−Butyrobetaine Hydroxylase 1	−3.296616849	3.01E−15	Down
SRD5A2	Steroid 5 Alpha−Reductase 2	−2.015257394	8.89E−17	Down
ACSM3	Acyl−CoA Synthetase Medium Chain Family Member 3	−2.028393911	5.43E−14	Down
GBA3	Glucosylceramidase Beta 3 (Gene/Pseudogene)	−2.463556707	1.26E−10	Down
CYP2B6	Cytochrome P450 Family 2 Subfamily B Member 6	−2.434282425	7.19E−12	Down
GLYAT	Glycine−N−Acyltransferase	−2.195290004	4.40E−10	Down
ZG16	Zymogen Granule Protein 16	−2.120752216	8.68E−17	Down
GLS2	Glutaminase 2	−2.400286201	3.28E−11	Down
MFSD2A	MFSD2 Lysolipid Transporter A, Lysophospholipid	−2.316298201	9.18E−14	Down
HAO2	Hydroxyacid Oxidase 2	−2.620033332	3.14E−12	Down
CYP2A6	Cytochrome P450 Family 2 Subfamily A Member 6	−2.322074035	4.79E−09	Down
CYP2A7	Cytochrome P450 Family 2 Subfamily A Member 7	−2.024761409	3.87E−11	Down
GYS2	Glycogen Synthase 2	−2.291074305	2.76E−08	Down
C3P1	Complement Component 3 Precursor Pseudogene	−2.088864100	4.97E−10	Down

LPA	Lipoprotein(A)	−2.368064154	2.20E−14	Down
AKR1D1	Aldo−Keto Reductase Family 1 Member D1	−2.557761216	4.72E−09	Down
GHR	Growth Hormone Receptor	−2.282112510	4.99E−11	Down
BCHE	Butyrylcholinesterase	−2.275286332	1.24E−08	Down
SULT1E1	Sulfotransferase Family 1E Member 1	−2.116352834	1.41E−12	Down
CNTN3	Contactin 3	−2.416032942	1.01E−08	Down
VNN1	Vanin 1	−2.262903436	2.18E−08	Down
CLRN3	Clarin 3	−3.124813776	2.61E−16	Down
CYP39A1	Cytochrome P450 Family 39 Subfamily A Member 1	−2.651808560	1.73E−12	Down
FREM2	FRAS1 Related Extracellular Matrix 2	−2.369084568	1.58E−11	Down
IGF1	Insulin Like Growth Factor 1	−2.348748467	4.12E−13	Down
MT1M	Metallothionein 1M	−2.834980961	4.68E−10	Down
CXCL2	C−X−C Motif Chemokine Ligand 2	−2.065043876	1.63E−12	Down
CD5L	CD5 Molecule Like	−2.054764444	1.33E−16	Down
FAM65C	RIPOR Family Member 3	−2.459200521	2.76E−22	Down
CRHBP	Corticotropin Releasing Hormone Binding Protein	−2.999371683	1.19E−24	Down
CLEC1B	C−Type Lectin Domain Family 1 Member B	−3.135877707	7.65E−30	Down
FCN3	Ficolin 3	−3.341982552	3.35E−23	Down
OIT3	Oncoprotein Induced Transcript 3	−3.229522436	1.19E−26	Down
PLAC8	Placenta Associated 8	−2.065061375	1.78E−20	Down
CXCL14	C−X−C Motif Chemokine Ligand 14	−3.978631486	1.53E−32	Down
HHIP	Hedgehog Interacting Protein	−2.803161676	1.78E−28	Down
CXCL12	C−X−C Motif Chemokine Ligand 12	−2.786652251	6.57E−17	Down
LIFR	LIF Receptor Subunit Alpha	−2.400421259	1.32E−18	Down
HAMP	Hepcidin Antimicrobial Peptide	−4.160273583	9.72E−18	Down
HGFAC	HGF Activator	−2.827246907	4.24E−18	Down
CDHR2	Cadherin Related Family Member 2	−2.446512749	2.71E−28	Down
IGFBP3	Insulin Like Growth Factor Binding Protein 3	−2.075588382	3.11E−19	Down
FOSB	FosB Proto−Oncogene, AP−1 Transcription Factor Subunit	−2.429478842	5.44E−15	Down
FOS	Fos Proto−Oncogene, AP−1 Transcription Factor Subunit	−2.191916691	5.23E−14	Down
PGLYRP2	Peptidoglycan Recognition	−2.162003459	5.42E−11	Down

	Protein 2			
APOF	Apolipoprotein F	−2.685010309	5.08E−14	Down
KMO	Kynurenine 3−Monooxygenase	−2.094420506	1.05E−14	Down
BCO2	Beta−Carotene Oxygenase 2	−2.333059189	3.39E−15	Down
CYP1A2	Cytochrome P450 Family 1 Subfamily A Member 2	−3.353119355	2.03E−19	Down
NAT2	N−Acetyltransferase 2	−2.481342467	2.86E−17	Down
DNASE1L3	Deoxyribonuclease 1 Like 3	−2.210444745	4.13E−15	Down
LCAT	Lecithin−Cholesterol Acyltransferase	−2.519286270	8.29E−23	Down
ZGPAT	Zinc Finger CCCH−Type And G−Patch Domain Containing	−2.195422544	1.05E−25	Down
KCNN2	Potassium Calcium−Activated Channel Subfamily N Member 2	−3.990836923	9.48E−28	Down
LINC01093	Long Intergenic Non−Protein Coding RNA 1093	−4.178427405	1.27E−24	Down
IDO2	Indoleamine 2,3−Dioxygenase 2	−3.321683185	1.20E−21	Down
IGFALS	Insulin Like Growth Factor Binding Protein Acid Labile Subunit	−2.047566911	6.13E−31	Down
CLEC4M	C−Type Lectin Domain Family 4 Member M	−2.871704973	1.60E−26	Down
FCN2	Ficolin 2	−3.636464429	2.08E−26	Down
CLEC4G	C−Type Lectin Domain Family 4 Member G	−3.162168263	6.20E−26	Down
CYP26A1	Cytochrome P450 Family 26 Subfamily A Member 1	−2.721213193	1.02E−24	Down
CNDP1	Carnosine Dipeptidase 1	−3.752327730	1.09E−22	Down
TTC36	Tetratricopeptide Repeat Domain 36	−2.957724000	3.21E−20	Down
TMEM27	Collectrin, Amino Acid Transport Regulator	−3.412451340	3.44E−20	Down
SLC25A47	Solute Carrier Family 25 Member 47	−2.179932568	3.05E−18	Down
IGHM	Immunoglobulin Heavy Constant Mu	−2.288931297	8.38E−17	Down
IGLC1	Immunoglobulin Lambda Constant 1	−2.299163764	2.05E−10	Down
IGLV1−44	Immunoglobulin Lambda Variable 1−44	−2.123044363	3.52E−10	Down
TACSTD2	Tumor Associated Calcium Signal Transducer 2	−2.189690710	1.42E−10	Down
CDH19	Cadherin 19	−2.201681135	8.57E−16	Down
PDGFRA	Platelet Derived Growth Factor	−2.360296332	1.63E−10	Down

	Receptor Alpha			
C7	Complement C7	−2.881514054	6.25E−13	Down
DCN	Decorin	−2.512236660	1.42E−11	Down
SRPX	Sushi Repeat Containing Protein X−Linked	−2.808193819	2.90E−18	Down
HGF	Hepatocyte Growth Factor	−2.710907042	3.06E−17	Down
WDR72	WD Repeat Domain 72	−2.008030745	7.80E−09	Down
KANK4	KN Motif And Ankyrin Repeat Domains 4	−2.230789907	9.14E−17	Down
ENO3	Enolase 3	−2.179728417	1.64E−17	Down
