# Supplementary material for: Significance of Identifying Key Genes Involved in HBV-Related Hepatocellular Carcinoma for Primary Care Surveillance of Patients with Cirrhosis
Source: Genes (Basel). 2022 Dec 10;13(12):2331. doi: 10.3390/genes13122331 (PMC9778294; doi:10.3390/genes13122331)
Supplement: Supplementary file 1 [file genes-13-02331-s001.zip › Table S2. The top 20 Hub genes identified by the plugin CytoHubba in Cytoscape software.pdf]

Table S2. The top 20 Hub genes identified by the plugin CytoHubba in Cytoscape software.

Gene symbol	Gene description	Degree
SPP1	Secreted Phosphoprotein 1	9
HMMR	Hyaluronan Mediated Motility Receptor	13
PBK	PDZ Binding Kinase	13
RRM2	Ribonucleotide Reductase Regulatory Subunit M2	13
CCNB1	Cyclin B1	15
CDK1	Cyclin Dependent Kinase 1	15
ASPM	Assembly Factor For Spindle Microtubules	15
DTL	Denticleless E3 Ubiquitin Protein Ligase Homolog	13
NEK2	NIMA Related Kinase 2	13
ECT2	Epithelial Cell Transforming 2	13
RACGAP1	Rac GTPase Activating Protein 1	13
PRC1	Protein Regulator Of Cytokinesis 1	13
TOP2A	DNA Topoisomerase II Alpha	14
ANLN	Anillin, Actin Binding Protein	13
BUB1B	BUB1 Mitotic Checkpoint Serine/Threonine Kinase B	13
HGF	Hepatocyte Growth Factor	9
CXCL12	C−X−C Motif Chemokine Ligand 12	9
DCN	Decorin	7
ESR1	Estrogen Receptor 1	15
IGF1	Insulin Like Growth Factor 1	12
