# Supplementary figures and images for: Significance of Identifying Key Genes Involved in HBV-Related Hepatocellular Carcinoma for Primary Care Surveillance of Patients with Cirrhosis
Source: Genes (Basel). 2022 Dec 10;13(12):2331. doi: 10.3390/genes13122331 (PMC9778294; doi:10.3390/genes13122331)

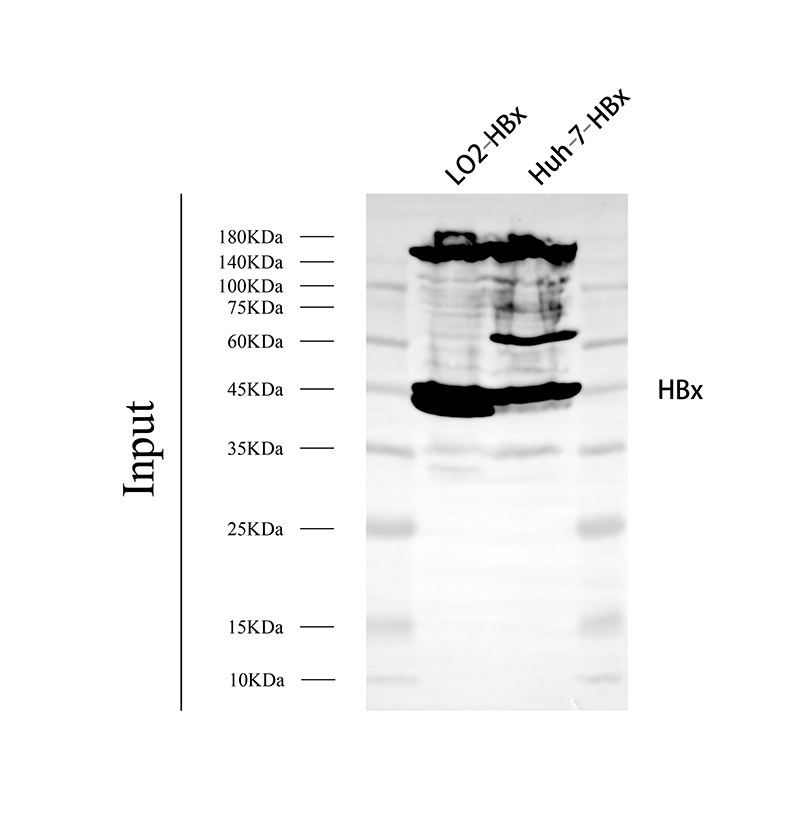

Supplement: Supplementary file 1 [file genes-13-02331-s001.zip › Figure S1.tif]

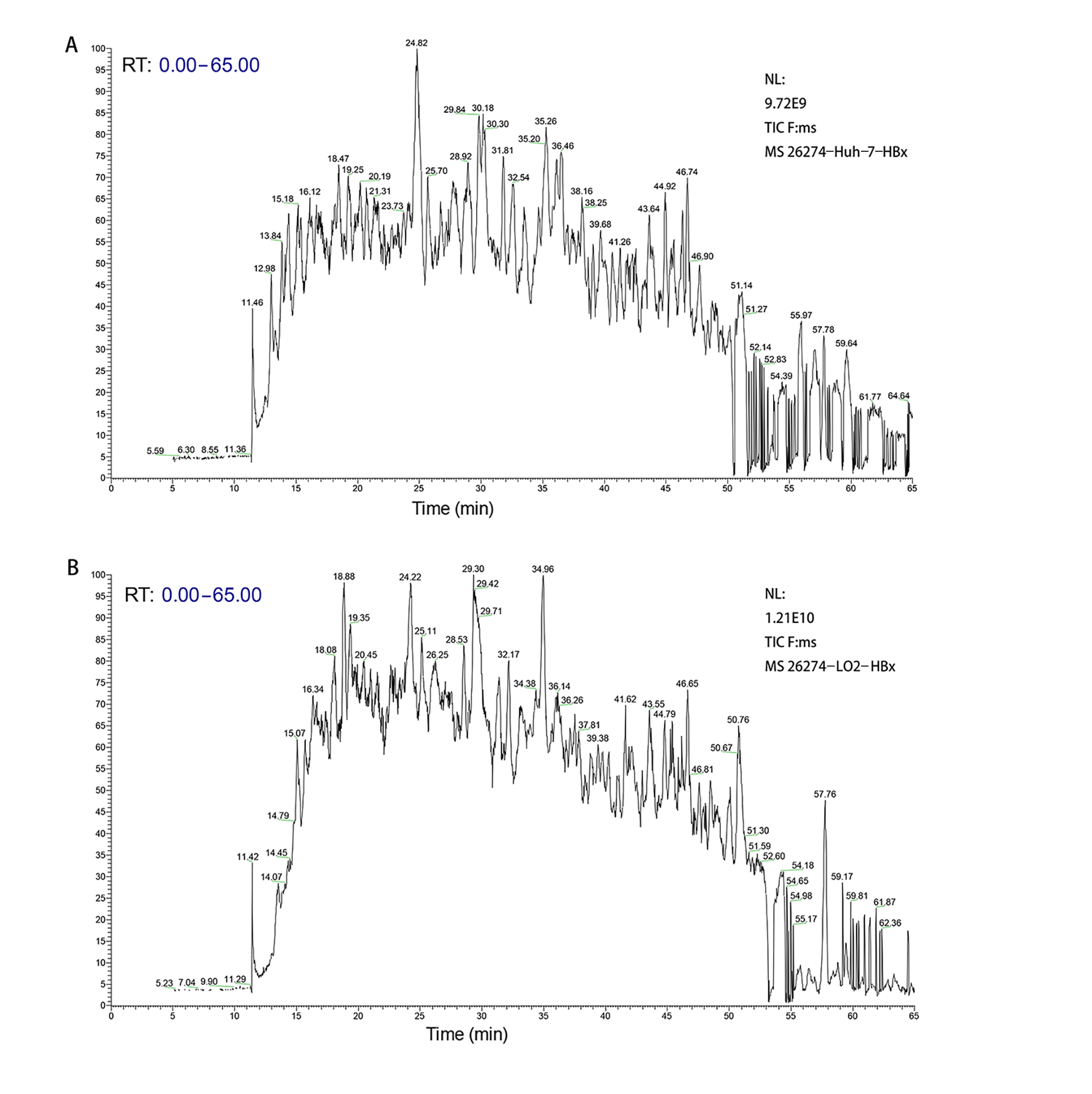

Supplement: Supplementary file 1 [file genes-13-02331-s001.zip › Figure S2.tif]
